# London Lancet

**Published:** 1858-05

**Authors:** 


### LONDON LANCET.

This venerable and valuable journal comes to us for April, from Messrs. Stringer & Townsend, New York, filled with choice articles from the pens of the ablest living writers. It is republished in this country at 222 Broadway, N.Y., for the extremely low price of $5 per year. We know of no one journal that will so many times (for we think there are none but what more than does it) pay the subscription price as this.

